# Beyond generative models: superfast traversal, optimization, novelty, exploration and discovery (STONED) algorithm for molecules using SELFIES†

**DOI:** 10.1039/d1sc00231g

**Published:** 2021-04-20

**Authors:** AkshatKumar Nigam, Robert Pollice, Mario Krenn, Gabriel dos Passos Gomes, Alán Aspuru-Guzik

**Affiliations:** Department of Computer Science, University of Toronto Canada alan@aspuru.com; Department of Chemistry, University of Toronto Canada; Vector Institute for Artificial Intelligence Toronto Canada; Lebovic Fellow, Canadian Institute for Advanced Research (CIFAR) 661 University Ave Toronto Ontario M5G Canada

## Abstract

Inverse design allows the generation of molecules with desirable physical quantities using property optimization. Deep generative models have recently been applied to tackle inverse design, as they possess the ability to optimize molecular properties directly through structure modification using gradients. While the ability to carry out direct property optimizations is promising, the use of generative deep learning models to solve practical problems requires large amounts of data and is very time-consuming. In this work, we propose STONED – a simple and efficient algorithm to perform interpolation and exploration in the chemical space, comparable to deep generative models. STONED bypasses the need for large amounts of data and training times by using string modifications in the SELFIES molecular representation. First, we achieve non-trivial performance on typical benchmarks for generative models without any training. Additionally, we demonstrate applications in high-throughput virtual screening for the design of drugs, photovoltaics, and the construction of chemical paths, allowing for both property and structure-based interpolation in the chemical space. Overall, we anticipate our results to be a stepping stone for developing more sophisticated inverse design models and benchmarking tools, ultimately helping generative models achieve wider adoption.

## Abstract 2

Interpolation and exploration within the chemical space for inverse design.

## Graphical abstract

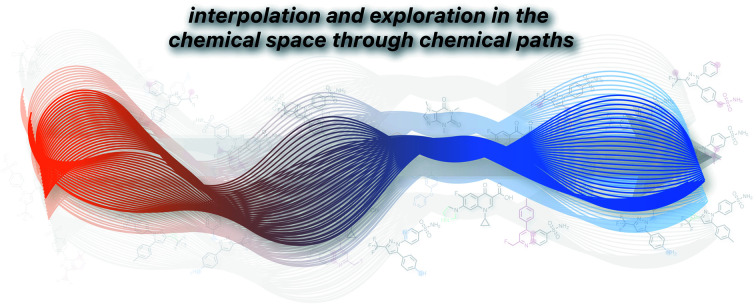

## I. Introduction

Generative models are a class of techniques with applications in inverse molecular design.^1^ Among them, variational autoencoders (VAEs),^2,3^ generative adversarial networks (GANs),^4,5^ recurrent neural networks (RNNs),^6,7^ deep reinforcement learning (DRL)^8,9^ and genetic algorithms (GAs)^10–17^ have been applied to the design of molecules. They can be roughly divided into models that aspire to produce only sensible molecules, either *via* learned or imposed structure generation procedures, and models that produce any structure satisfying basic valence rules. Notably, for practical purposes, the latter class of models requires additional filters to remove unstable, reactive or toxic moieties before further evaluation. Importantly, the choice of molecular representation employed in these approaches impacts performance dramatically. Deep generative models trained on molecular representations form low dimensional latent spaces enabling the sampling of unseen molecules. This allows for exploration in the chemical space and interpolation by chemical path formation^3^ using geometric operations in the continuous latent spaces. In contrast to typical implementations of genetic algorithms with the SMILES string representation,^18,19^ a unique aspect of these deep learning techniques is that the generation of new molecules does not require the design of hand-crafted rules. However, they can require access to large datasets, either labeled or unlabeled depending on the specific task at hand, and expensive computational resources to offset large training times. Furthermore, with fragile representations such as SMILES, large areas of a latent space can correspond to invalid molecules.^3^ Alternatively, deep generative models using molecular graphs represented as adjacency matrices have also been demonstrated with applications in drug design.^20,21^ Recently, the development and application of a 100% valid strings representation – SELFIES^22^ – has been demonstrated for inverse design.^23^ Compared to SMILES and adjacency matrices, the use of SELFIES in generative models overcomes the problem of generating invalid molecules.

In this work, using SELFIES as a robust molecular representation, we propose an efficient set of algorithms (STONED) to perform exploration and interpolation in the chemical space (Section II A). These tasks are commonly addressable by expensive deep generative models or stochastic optimization approaches like evolutionary methods.^24^ Our algorithm avoids the need for extensive training times, large datasets, and hand-crafted rules for obtaining novel molecules, and allows to interpolate deterministically between molecules. We achieve this *via* string manipulations of SELFIES and demonstrate the ability to form local chemical subspaces (Section II B), allowing for local optimization, and obtain chemical paths (Section II C), enabling interpolation between structures. Additionally, we demonstrate applications in designing molecules for material science (Section II D) and drug development (Section II C 2). On established benchmarks, our algorithm achieves non-trivial results despite not using any sophisticated optimization engines and is comparable in its capabilities to the state of the art in generative modeling. The ease of obtaining molecules for local optimization and interpolation *via* chemical paths allows for our methods to be used in high-throughput virtual screening for materials science,^25^ catalysis,^26^ and drug design.^27^ Ultimately, we anticipate that our results will stimulate more powerful models, more meaningful benchmarks, and more widespread use of generative models in general.

## II. Results and discussion

### A. Algorithmic overview

**Fig. 1:**
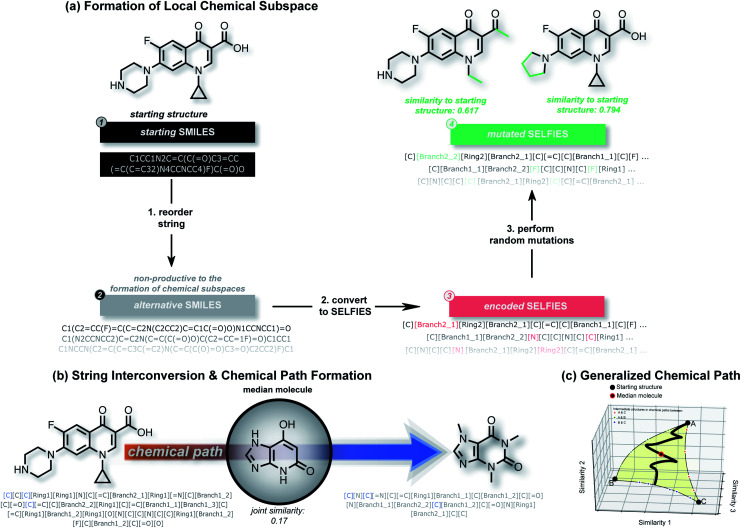
Illustration of string manipulations within STONED to form local chemical subspaces (a, see Section II B) for a given structure, discovering median molecules on the chemical path between two structures (b, see Section II C) and formation of generalized chemical paths between more than two molecules (c, see Section II D).

In this work, we show that modifications within the SELFIES molecular representation are a powerful tool for performing structural and property-based changes to molecules. Akin to deep generative models, these changes can be utilized for forming local chemical subspaces of molecules (Fig. 1(a)), forming chemical paths between known molecules (Fig. 1(b and c)) and obtaining a molecule representative of multiple structures (median molecules – Fig. 1(b)). For that purpose, we introduce STONED, a set of algorithms where a single step of molecular generation is carried out, that are optionally based on initial seed structures. Each of these algorithms makes use of incremental changes within the SELFIES representation of a molecule. Currently, we make use of four important techniques within STONED. Firstly, in SELFIES, random character changes always correspond to valid molecules. Unlike other molecular representations, this allows us to perform random changes to molecules without subsequent validity checks. Moreover, we demonstrate that the position of the random character changes can be used as hyperparameter to switch between exploration and exploitation in the molecules generated. Secondly, every molecule can be represented with multiple SMILES strings, and multiple corresponding SELFIES. Since a single SELFIES has a limited number of possible character changes, we enhance the diversity of the generated structures by generating and utilizing multiple representations for the same molecule. Without the use of reordering before making changes, the number of generated structures is severely limited. Thirdly, we demonstrate that interpolations between an arbitrary number of reference molecules can be performed deterministically by matching SELFIES characters at equivalent positions between the reference strings. Lastly, we use the efficiency of fingerprint comparisons as a tool to enforce structural similarity because edit distances within SELFIES do not reflect it. With these techniques, we can form local chemical subspaces, discover median molecules and form chemical paths for structural interpolation.

### B. Formation of local chemical spaces

The ability to generate the structural neighborhood of known molecules allows for local optimization. In drug discovery, candidate libraries are typically designed based on similarity to known active compounds aiming for further property improvements.^28,29^ Usually, the formation of these local chemical subspaces is achieved with predefined rules.^24,30,31^ However, the design of domain-specific rules for structure modification is time-consuming, non-trivial, and application-dependent. Hence, systematic methods for forming local chemical subspaces with minimal bias that can be used for any class of molecules are important. Additionally, on-the-fly structure generation has recently been considered as a benchmark to evaluate generative molecular design models in GuacaMol^32^ and MOSES.^33^ In these benchmarks, model quality is determined by the number of unique molecules generated within predefined fingerprint similarity thresholds. Notably, for deep generative models, the generation of unique molecules close to a target is biased by the resemblance between molecules of an independent training dataset and the target structure.

**Fig. 2:**
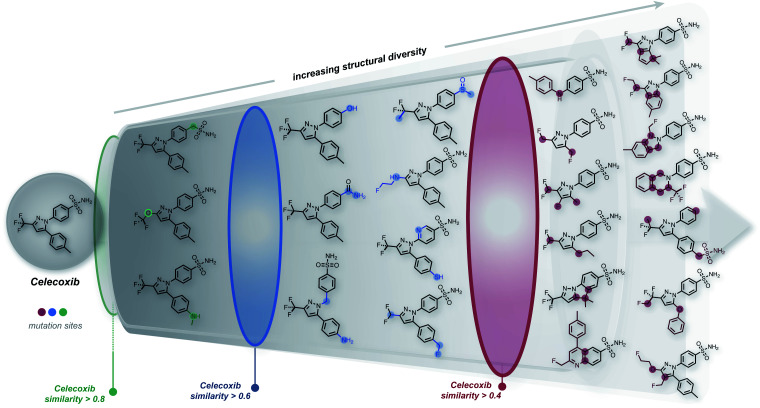
Systematic local chemical space exploration of celecoxib using mutations of different SELFIES representations. The similarity is calculated using the Tanimoto distance of the ECFP4 fingerprint between celecoxib and the generated structures.

We started this work by performing point mutations of the molecules aripiprazole, albuterol, and mestranol^32^ in the SELFIES representation to generate local chemical subspaces. A point mutation in the SELFIES representation corresponds to a single character addition, deletion or replacement. As delineated in Table 1, STONED is able to generate vast local chemical subspaces requiring only one data point as a seed. Additionally, in comparison to the state of the art in deep generative modeling for molecular design, our algorithm is an order of magnitude faster. Notably, for each of these experiments, the respective fingerprints suggested in the analogous GuacaMol benchmarks were used. Fig. 2 illustrates the ability of our algorithm to generate diverse structures in the neighborhood of the known drug celecoxib.^34^ As expected, we observe that the fraction of unique molecules obtained decreases with more stringent structure-based fingerprint similarity requirements. Importantly, this is a general feature of the SELFIES representation. As depicted in Fig. S2 (left),† mutating molecules randomly in the SELFIES representation rarely preserves high molecular similarity. Additionally, molecular similarity to the initial structure, on average, decreases with the number of mutations performed which is intuitive.

**Table 1:** Number and percentage of unique molecules obtained within different fingerprint-based similarity thresholds (δ) of the starting structures. The molecules in each experiment were generated from 250 000 random string mutations of the starting structures. Additionally, for celecoxib, we also formed the local chemical space with a scaffold constraint

Starting structure (method)	Fingerprint	Number of molecules (and percentage)		
		*δ* > 0.75	*δ* > 0.60	*δ* > 0.40
Aripirazole (SELFIES, random)	ECFP4	513 (0.25%)	4206 (2.15%)	34 416 (17.66%)
Albuterol (SELFIES, random)	FCFP4	587 (0.32%)	4156 (2.33%)	16 977 (9.35%)
Mestranol (SELFIES, random)	AP	478 (0.22%)	4079 (1.90%)	45 594 (21.66%)
Celecoxib (SELFIES, random)	ECFP4	198 (0.10%)	1925 (1.00%)	18 045 (9.44%)
Celecoxib (SELFIES, terminal 10%)	ECFP4	864 (2.02%)	9407 (21.99%)	34 187 (79.91%)
Celecoxib (SELFIES, central 10%)	ECFP4	111 (0.08%)	1767 (1.32%)	15 348 (11.45%)
Celecoxib (SELFIES, initial 10%)	ECFP4	368 (0.53%)	7345 (10.53%)	34 702 (49.74%)
Celecoxib (SMILES, random)	ECFP4	122 (18.43%)	515 (77.49%)	662 (100.00%)
Celecoxib (SMILES, terminal 10%)	ECFP4	90 (20.79%)	368 (84.99%)	433 (100.00%)
Celecoxib (SMILES, central 10%)	ECFP4	114 (22.18%)	419 (81.52%)	514 (100.00%)
Celecoxib (SMILES, initial 10%)	ECFP4	122 (19.71%)	490 (79.16%)	619 (100.00%)
Celecoxib (DeepSMILES, random)	ECFP4	132 (4.43%)	953 (31.99%)	2793 (93.76%)
Celecoxib (DeepSMILES, terminal 10%)	ECFP4	106 (9.73%)	513 (47.11%)	1083 (99.45%)
Celecoxib (DeepSMILES, central 10%)	ECFP4	53 (6.54%)	162 (19.98%)	658 (81.13%)
Celecoxib (DeepSMILES, initial 10%)	ECFP4	105 (9.28%)	609 (53.80%)	1106 (97.70%)
Celecoxib (SELFIES, scaffold constraint)	ECFP4	354 (0.44%)	6311 (7.79%)	53 479 (66.07%)
Celecoxib (CReM, ChEMBL: SCScore ≤ 2.5)	ECFP4	239 (0.58%)	5547 (13.47%)	14 887 (36.14%)

While the success rate of mutations leading to structurally similar molecules is relatively low (Table 1), our approach is extremely efficient, with the entire experiment running in just a few minutes on an ordinary laptop at the time of writing (Intel i7-8750H CPU, 2.20 GHz). In particular, the most time-consuming benchmark in Table 1 was the formation of the subspace of aripiprazole, completing in 500 seconds. The most expensive step in this experiment involved performing multiple SELFIES mutations and subsequently converting all mutated strings into SMILES, taking 400 seconds. Importantly, this step can be made more efficient by conducting mutations on different strings using parallel workers. Hence, this algorithm possesses extensive parallelizability. For comparison, using the same setup, we also formed the local chemical subspace of celecoxib using either SMILES or DeepSMILES. For SMILES, merely 0.30% of the mutated structures corresponded to valid molecules. With DeepSMILES, merely 1.44% of the mutated structures were valid. In addition, we observed that random mutations within SMILES and DeepSMILES led to lower structural diversity compared to SELFIES (see Table 1). Additionally, we also formed the chemical subspace of celecoxib while preserving a pre-selected scaffold (see celecoxib (SELFIES, scaffold constraint) in Table 1). Discarding all mutated strings that do not contain the scaffold, *i.e.*, keeping only 2.8% of all mutated strings, STONED readily proposed a large number of structures in the neighborhood of celecoxib. Overall, the speed and scalability of our methods suggest that it can be readily applied to extend datasets used in machine learning for creating more robust generalizable models.

Importantly, we also found that a general strategy for preserving molecular similarity during random SELFIES mutations of the starting structure is to restrict the location of the SELFIES changes. Restricting the mutations to either the initial or the terminal characters yields mutated structures that are more similar to the initial structure than when the mutation position is either chosen randomly or restricted to the middle characters (see Table 1 and Fig. S3;† initial, central or terminal 10%). It should be emphasized that this is not just a curious finding but can be used systematically to choose between exploration and exploitation for the structural space generated using STONED. In addition, it can be employed in conjunction with scaffold constraints as restricting the mutations to the terminal 10% of the SELFIES also increases the probability to retain scaffolds. We repeated the scaffold constraint experiment from above but restricted the mutations to the terminal 10% and found that 36.3% of all mutated strings retained the scaffold, which corresponds to a more than 10-fold increase in the scaffold retention rate. Notably, trying to use the same type of character mutation restriction for SMILES or DeepSMILES does not provide the same kind of tunability between exploitation and exploration of the generated structures. As additional comparison to alternative methods, we also generated the local chemical subspace of celecoxib using the recently developed expert system CReM, a fragment-based approach.^35^ Taking fragments and mutation rules from a subset of ChEMBL^36,37^ with an SCScore ≤ 2.5, CReM generates significantly more structures in the structural neighborhood than fully random SELFIES mutations but less than when SELFIES mutations are restricted to the terminal characters. This shows that STONED is comparable in performance to expert systems like CReM.

Notably, in the experiments described above, we performed mutations solely on the starting structure. A natural extension is to repeat the procedure on all distinct neighbours, *i.e.*, molecules produced by point mutations from the initial structure, thereby extending the subspace search significantly. To demonstrate the power of this approach, starting from the randomly mutated structures of celecoxib, we repeated the random mutations on all unique molecules obtained in the first step. Consequently, we generated more than 17 million unique molecules, 120 thousand of which have a similarity greater than 0.4 with respect to celecoxib (see Fig. S4†) showing that the structural coverage of the local subspace can be increased immensely by including structural next nearest neighbors of the initial seed molecule.

**Fig. 3:**
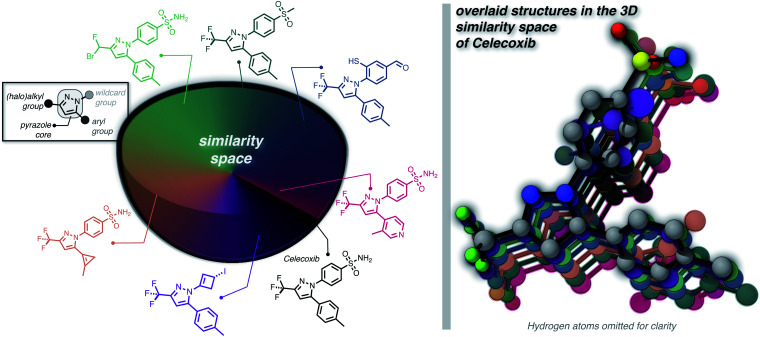
Systematic local exploration of the 3D similarity space of celecoxib.

Furthermore, we wanted to demonstrate the full potential of the chemical subspace exploration by replacing the ECFP4 fingerprint similarity with 3D fingerprints to form geometry-based chemical subspaces. To do that, we generated conformers of celecoxib and 2350 of its mutants with RDKit using the implemented conformer ensemble routine. The lowest-energy conformer was selected and the 3D similarity between the structures of celecoxib and its mutants was estimated using the E3FP similarity metric.^38^ Consequently, we found 206 structures with an E3FP similarity larger than 0.2, 31 of which were even larger than 0.3. Selected structures are depicted in Fig. 3 with an overlay of the corresponding conformers with the structure of celecoxib. This shows that generating the 3D similarity space with STONED and E3FP similarity is straightforward allowing it to be applied to structure-based inverse design. Notably, we hypothesize that the 2D or 3D structure-based fingerprints can also be replaced with efficient property-based molecular descriptors^39–41^ for systematic property space exploration in an analogous way.

### C. Properties along chemical paths

#### 1. Measuring joint molecular similarity

A median molecule of a given set of reference molecules is a molecule that resembles all the reference molecules simultaneously based on a selected similarity metric.^42^ Recently, the generation of median molecules has been proposed as a benchmarking objective within GuacaMol.^32,43^ In this benchmark, termed the median molecule discovery objective, the goal is to maximize the similarity to a predefined set of structures simultaneously, *i.e.*, the joint molecular similarity. The problem can be viewed as identifying the largest fragments that are identical in a set of molecules. Notably, when the mutual similarity between the reference structures is small, the generation of median molecules can be challenging leading to low joint similarity metrics.

Importantly, the similarity of proposed median structures to the references can be gauged *via* structure-based fingerprint similarity measures. In GuacaMol, a median molecule (*i.e.*, m) of two known structures (*i.e.*, *m*′, *m*^′′^) is assessed based on the geometric mean of the respective fingerprint similarities to the two reference structures. The higher the geometric mean, the better the median molecule. However, we observe that maximizing the geometric mean of fingerprint similarities does not capture joint molecular similarity satisfactorily. The metric can return large values despite possessing high similarity only to one structure (see Section S2†). Therefore, we propose to redefine joint similarity *F*(*m*) for an arbitrary number of reference molecules *M* = {*m*_1_, *m*_2_, …}; *n* = |*M*|, which is discussed in detail in the ESI (Section S2),† as follows, to penalize higher similarities to only a subset of the reference molecules more severely:1
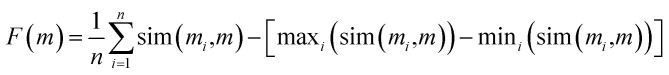


In the subsequent sections, we investigate the behaviour of this joint molecular similarity along chemical paths between molecules which inadvertently leads to the generation of median molecules.

#### 2. Interpolation *via* chemical path formation

Chemical paths are series of molecules where each successive member is increasingly similar to the target.^44^ Motivated by the rediscovery benchmark objective in GuacaMol,^32^ which can be interpreted as the formation of chemical paths between the seed structures and the desired target molecule, we explored the possibility to use the robustness of SELFIES for deterministic molecular interpolation. Within the SELFIES universe, *i.e.*, the set of all strings composed of SELFIE characters, the notion of path formation has a unique formulation. Using character replacements, deletions, and additions as possible mutations, for any given pair of SELFIES representing two distinct molecules, a finite number of modifications exist that interconvert them. This interconversion can be performed deterministically by simply comparing the SELFIES characters at equivalent positions in the two strings and successively changing the characters of the initial molecule to the characters of the target molecule. We define every successive molecule encountered in this transformation as within a path. Every one of these mutated SELFIES corresponds to a valid molecule. While this deterministic interconversion can in principle be achieved with any string-based molecular representation like SMILES or DeepSMILES,^45^ most of these modifications will very likely lead to the formation of syntactically or semantically invalid molecules.^22^ Hence, there will be specific islands of valid molecules embedded within a sea of invalid strings. For instance, between the SMILES strings CCC1CCC1CCC and CCCCCCCCC, no single mutation that corresponds to an increase in Levenshtein similarity forms valid molecules leading to a string without a valid chemical structure in the corresponding path. Accordingly, previous approaches based on string representations like SMILES made use of stochastic structural interpolation between structures using evolutionary algorithms^24^ or performed geometric interpolation in latent spaces of deep generative models.^3^

**Fig. 4:**
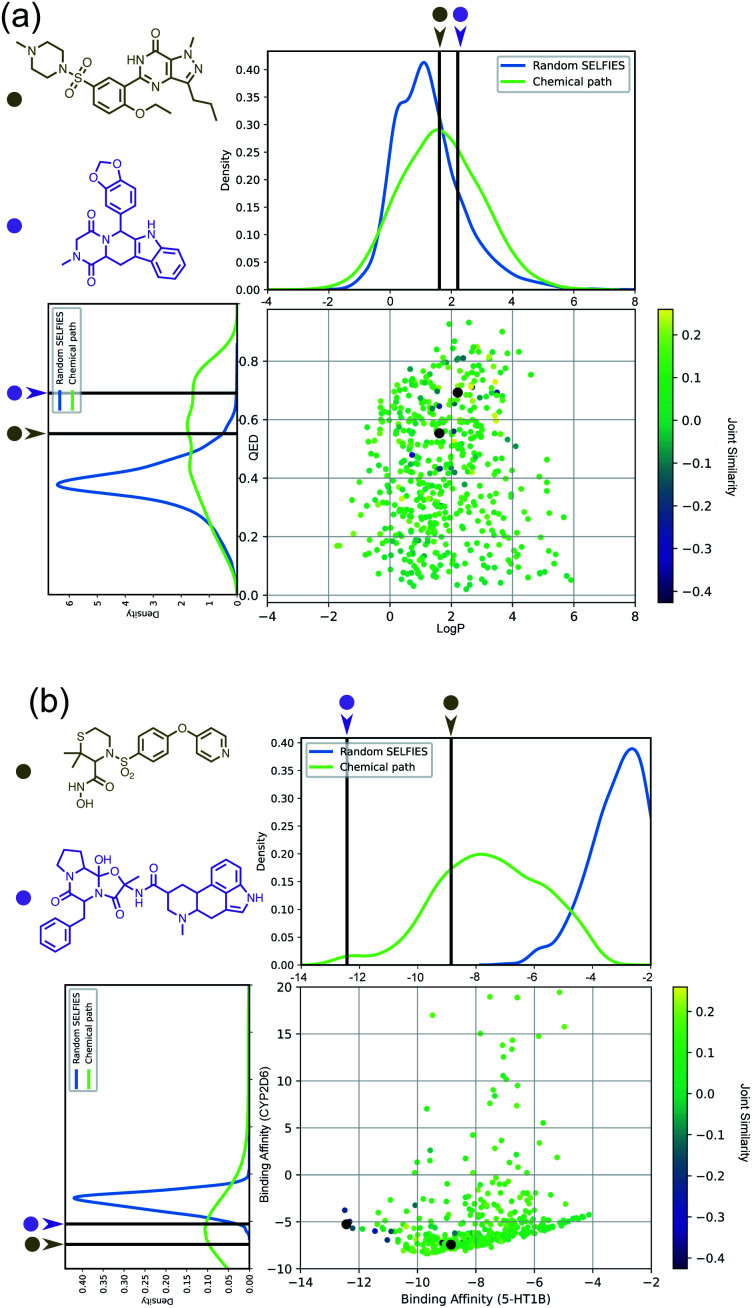
(a) log *P* and QED values of molecules encountered along chemical paths between tadalafil and sildenafil. (b) Ligand binding affinities of molecules encountered along chemical paths between dihydroergotamine and prinomastat. For both subfigures, the corresponding reference properties are indicated by black lines.

**Fig. 5:**
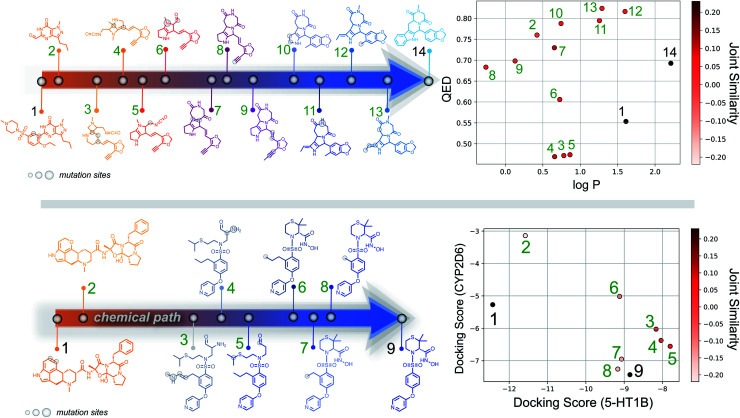
(Top) Example of molecules along a chemical path between tadalafil and sildenafil, with their corresponding log *P* and QED scores. (Bottom) Example of a chemical path between dihydroergotamine (binder for 5-HT1B) and prinomastat (binder for CYP2D6). Docking scores for the intermediate structures on both proteins and their joint similarity to the reference structures are provided in the diagram to the right.

Importantly, while a monotonically increasing fingerprint similarity score is not observed along paths generated deterministically between two SELFIES, one can extract chemical paths by requiring fingerprint similarities to increase and removing all structures that lead to similarity drops. Compared to generating chemical paths using SELFIES stochastically, by making use of evolutionary algorithms, our deterministic approach leads to a speedup of more than one order of magnitude. To avoid holes in the beginning of the chemical paths, we imposed the requirement for increasing fingerprint similarities only after the first point mutation of the starting structure. Because of the speed and parallelizability of this chemical path generation method, motivated by the idea that similarity in structure can correspond to similarity in properties, we looked into properties of molecules along chemical paths. As an initial test, we considered the water–octanol partition coefficient (log *P*)^46^ and the quantitative estimate of drug-likeness (QED)^47^ in paths between the known drugs tadalafil and sildenafil (Fig. 4(a)), as estimated using RDKit.^48^ One of these chemical paths is shown in Fig. 5 (top), and the similarities to the starting and target structures along the path as well as the comparison of the corresponding geometric mean joint similarities and our newly defined joint similarities are illustrated in Fig. S6 (top).† These results demonstrate that the redefined joint similarity is more reliable for indicating molecules that are similar to several reference structures simultaneously.

Moreover, we analyzed the binding affinity estimated *via* docking^49^ in chemical paths between dihydroergotamine and prinomastat as a more challenging type of property to optimize (Fig. 4(b)). Dihydroergotamine and prinomastat have been discussed in the literature as potential inhibitors for the protein structures of serotonin (5-HT1B)^50^ and P450 2D6 (CYP2D6).^51^ The 5-HT1B receptor is a target for antimigraine drugs such as ergotamine and dihydroergotamine.^50^ P450 2D6, on the other hand, contributes to the metabolism and elimination of more than 15% of the drugs used in clinical practice. Among individuals, considerable variations exist in the efficacy and amount of CYP2D6 enzyme production. As a result, a clinical drug dose may need to be altered to account for the metabolization speed of CYP2D6.^52^ Prinomastat, as an inhibitor, decreases enzyme production, thereby allowing increased efficacy of certain drugs. Our goal in this experiment is to find molecules encountered along the paths between dihydroergotamine and prinomastat that can simultaneously bind (*i.e.*, possess negative binding affinities large in magnitude) to both proteins (see Fig. 4(b)). One selected chemical path is depicted in Fig. 5 (bottom). Moreover, we also compared both the similarities to the reference molecules along the path and our redefined joint similarity metric to the corresponding geometric mean joint similarities (see Fig. S6 (bottom)†). These diagrams show again that the joint similarity introduced in this work avoids molecules that are similar to only one of the reference structures. For the selected path, the docked molecules are depicted in Fig. S7.† Notably, there are significant structural jumps along these chemical paths, *i.e.*, successive molecules show relatively large structure changes. They largely stem from the condition that every molecule along a chemical path needs to increase in similarity to the target. Accordingly, molecules along the full path that led to a decrease in similarity to the target after the first mutation were removed causing these large changes in the remaining molecules. Notably, some of the molecules obtained have unstable functional groups or would tautomerize in solution to a different structure. To improve both their stability and synthetic feasibility, rules based on domain knowledge can be implemented to filter out structures that are infeasible.

Importantly, this experiment demonstrates the ability to perform efficient structural interpolation between molecules without the need to form continuous representations within deep generative models. Our simple algorithm for obtaining chemical paths possesses considerable potential for parallelization and does not need a large number of data points as input. Particularly, Cieplinski *et al.*^53^ noted that with realistic training set sizes (*i.e.*, consisting of a few thousand points), deep generative models have difficulty optimizing docking scores. In contrast, as illustrated in Fig. 4(b), our approach to forming chemical paths between two known ligands yields several structures with non-trivial binding affinities to both proteins without any optimization routine.

### D. Median molecules for photovoltaics

As pointed out previously, forming chemical paths between two molecules inadvertently leads to the generation of median molecules. Next, we generalized the concept of a chemical path to potentially having more than two reference molecules (see Fig. 1(c) and Section S3†). As an application example, we considered the organic photovoltaic dataset from the Harvard Clean Energy (HCE) project,^31^ and identified 100 sets of three molecules (referred to as triplets) so that the first has a high lowest unoccupied molecular orbital (LUMO) energy, the second a high dipole moment, and the third a high energy difference between the highest occupied molecular orbital (HOMO) and LUMO energies (HOMO–LUMO gap), while having low values for the respective other two properties. This choice of properties reflects potential design objectives for organic photovoltaics.^54^ HOMO–LUMO gap and LUMO energies determine the energy of light absorption and acceptor ability, respectively, while dipole moment can be considered a crude proxy for intermolecular interaction strength. We simulated these properties using the semiempirical GFN2-*x*TB quantum chemistry method^55^ (see details in the Methods Section†).

**Fig. 6:**
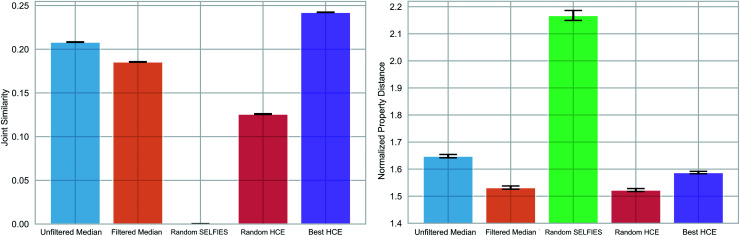
Multi-objective property optimization of potential molecules of interest for photovoltaics. Structural (left) and property similarity (right) of generated median molecules compared to specific sets of three molecules taken from the Harvard Clean Energy (HCE) database. Bar plots for the mean, and error bars for the standard deviation of the mean (2 standard deviations) are shown for the joint similarity and the normalized property distance of the 100 median structures with highest joint similarities to the references, with (*Filtered Median*) and without (*Unfiltered Median*) a bridgehead atom filter. They are compared to *Random SELFIES* and to molecules from the HCE database (*Random HCE* and *Best HCE*). The obtained median molecules are very close to *Best HCE* in joint similarity and slightly better in the properties.

We compared the ability of the obtained median molecules to resemble the triplet references in structure (Fig. 6 (left)) and property (Fig. 6 (right)). A selection of the generated median molecules is shown in Fig. S9.† Notably, higher joint similarities indicate that the median molecules resemble the triplets more closely in structure. However, low values of the normalized property distance indicate that the median molecules have properties closer to the respective reference structures. For each triplet identified from the HCE database, we used the 100 median molecules with the highest joint similarities to the reference structures from chemical paths between the three reference structures (*Unfiltered Medians*). We observed that many of these median molecules possessed bridgehead atoms with double bonds, a very unstable structural feature.^56^ To remedy this problem, we added a simple filter discarding molecules with bridgehead atoms in general (*Filtered Medians*).

In Fig. 6, *Random HCE* refers to sampling 100 random structures from the HCE database for each triplet, while *Best HCE* refers to the 100 molecules with the highest joint similarities to the reference structures available within the database. Importantly, we found that the median molecules are significantly closer in both structure and target properties to the respective triplets compared to *Random HCE*. In addition, they are also closer to the respective triplets in the investigated properties compared to *Best HCE* showing that generating median molecules can be an effective strategy for performing multi-objective property optimization (see Fig. S8 and Table S3† for detailed statistics). Importantly, this task is a complicated multi-objective optimization in a chemical subspace tailored for a very specific application. Our method is able to produce molecules that are similar in structure to three molecules simultaneously. In that regard, our method produces structures similar in both structural similarity and property compared to a database of molecules obtained using a building block approach based on expert knowledge. Hence, our results are very promising for fully automated exploration of chemical subspaces based on a few reference structures without defining building blocks and rules to construct molecules.

Expert rules-based systems can yield median molecules as well,^35,42,57,58^ but their use can be application-dependent. For example, a potential algorithm could disassemble the reference structures into fragments by breaking rotatable bonds and then recombine the fragments in a building block approach, akin to the design of CReM.^35^ However, this technique would not be generalizable to molecules without rotatable bonds, such as fused polyaromatics, and more sophisticated algorithms would be required. Our method differs in that it requires no expert knowledge and relies solely on the graph representation of molecules within SELFIES and necessarily leads to a median molecule. Deep generative models can be used to avoid such problems, with expert knowledge being derived solely from a known dataset. However, they require many training examples, potentially even labeled training data depending on the specific task at hand. In contrast, our approach is both rules-free and training-free.

## III. Comparison of molecule generation algorithms

Lastly, to compare the performance of STONED with alternative generative models, we performed the full set of GuacaMol benchmarks.^32^ As STONED does not require training, we simply identify the single best molecule in the provided training data for the corresponding benchmark task and use it to generate the surrounding local chemical subspace *via* SMILES reordering and SELFIES mutations. The resulting molecules are evaluated for their performance in the benchmark. Importantly, this one-shot optimization approach is able to compete with several of the state-of-the-art generative models having an overall GuacaMol score of 14.70 (see Table S4†). Furthermore, we also measured the compound quality of the molecules generated in the GuacaMol benchmarks as proposed in the literature.^32^ We find that 38% of all the top 100 molecules of each benchmark combined pass the quality filters, which is comparable to the performance of both Graph GA and SMILES GA.^32^

Finally, we compared the capabilities of STONED with established algorithms for generating molecules (Table 2). Similar to VAEs, GANs and RL approaches, STONED relies on random changes of molecules within a given representation superseding hard-coded expert rules.^14,16^ In contrast, expert systems (ES) typically incorporate fragment combination rules and heuristic synthesizability and stability checks.^16,24,31^ Moreover, as SELFIES covers the entire molecular space representable by molecular graphs, STONED allows the systematic exploration and generation of all these compounds. Importantly, neither of the alternative methods considered offer a comparable structure coverage. The hardcoded rules of ES tend to limit exploration, and within VAEs, GANs and RL the generated molecules have not been found to stray too far from the training set. Another important property we considered is interpolatability, *i.e.* the possibility to interpolate between two molecules deterministically. Interpolation in STONED is constrained by the number of distinct characters in the SELFIE string. VAEs and GANs can use geometric interpolation in the latent space. ES such as Molpher^16^ and the chemical space travel algorithm^24^ perform exploration and interpolation stochastically similar to a GA. RL techniques typically do not form a continuous representation, which limits their possibility for deterministic interpolation. Furthermore, VAEs, GANs and RL techniques are capable of property-based navigation, *i.e.*, selecting structural modifications that are likely to improve properties. This is often achieved *via* property estimators such as neural networks. In VAEs, prediction networks are often employed for arranging latent representations based on properties allowing gradient-based navigation in the property space. Both STONED and ES can be used in GAs for property-based navigation, but only in a stochastic way. Additionally, VAEs, GANs and RL models require training which can be prohibitive due to the potential need for multiple GPUs. STONED and ES, in comparison, do not require any training. Finally, VAEs, GANs and RL require considerably large training datasets. Contrarily, STONED and ES require very few, if any, reference points. To summarize, STONED combines the merits of both classical ES and more sophisticated ML methods for molecule generation closing a gap in the available methods to navigate the chemical space.

**Table 2:** Comparison of algorithms for the generation of molecules. ✓ and ✗ indicate the presence and absence of a feature, respectively. ∼ indicates that implementation of a feature within the algorithm is, in principle, possible but not straightforward or has not been carried out yet

Feature	ES	VAE	GAN	RL	STONED
Expert rule-free	✗	✓	✓	✓	✓
Structure coverage	∼	∼	∼	∼	✓
Interpolatability	✗	✓	✓	✗	✓
Property-based navigation	∼	✓	✓	✓	∼
Training-free	✓	✗	✗	✗	✓
Data independence	✓	✗	✗	✗	✓

## IV. Conclusion and outlook

In this work, we have introduced the STONED algorithms to perform simple and efficient exploration and interpolation in the chemical space. We demonstrate the simplicity of forming local chemical subspaces and obtaining chemical paths using SELFIES as molecular representation, readily generating a vast amount of molecules that are structurally similar to the seed structures. Furthermore, we redefine the joint molecular similarity to avoid bias towards only a subset of the reference structures and show that deterministic chemical path formation using STONED is an extremely efficient heuristic algorithm to find median molecules. Additionally, we showcase applications of STONED for molecular design in both drug discovery and materials science.

The speed, parallelizability, and performance of STONED suggests that it can be used for practical tasks such as high-throughput virtual screening.^59^ In optimization algorithms such as genetic algorithms, we believe that median molecule generation through our approach can be used as a general crossover rule. The current evaluation standard for deep generative modeling includes producing valid molecules that resemble specific datasets.^32,33^ With the guarantee of molecular validity in SELFIES by design, perfect results in the validity benchmark can be trivially achieved. Furthermore, we demonstrate the simplicity of generating multiple structures that resemble a known set of molecules. Among other benchmarks, properties such as penalized log *P* and QED do not represent the complexity of molecular design, making them an insignificant benchmarking objective. Accordingly, we also demonstrated application to more complicated multi-objective property optimizations including protein docking, dipole moments, LUMO energies and HOMO–LUMO gaps as target properties. By introducing STONED, a fast suite of algorithms that can compete reasonably with deep generative models on several recently introduced benchmarks, we believe that we provide a stepping stone to improve generative modeling for molecular design and its benchmarking.^60^

## V. Methods

### A. Formation of local chemical spaces

Starting from a single molecule, we obtain 50 000 SMILES orderings representing the same structure, convert all of them to the SELFIES representation, and perform 1–5 point mutations. Hence, a total of 250 000 strings are generated per experiment. A single mutation consists of a SELFIES character replacement, deletion, and addition at random positions of the string. This process is repeated to perform multiple mutated structures. All the mutants are subsequently converted back to SMILES for calculating their similarity to the original molecule based on various fingerprint similarity measures. Within Table 1, this process is repeated for: aripirazole, albuterol, mestranol, celecoxib (SELFIES, random), celecoxib (SELFIES, terminal 10%), celecoxib (SELFIES, central 10%), celecoxib (SELFIES, initial 10%) and celecoxib (SELFIES, scaffold constraint). For celecoxib (SMILES, random) and celecoxib (DeepSMILES, random), up to 5 random mutations are performed within the corresponding representations on 50 000 randomly ordered strings. For celecoxib (CReM, ChEMBL: SCScore ≤ 2.5), we performed the MUTATE and GROW operations, using a database of fragments provided in the CreM GitHub repository (vert replacements02_sc2.5.dbvert).^61^ The mutate and grow operations were applied to celecoxib both with and without explicit hydrogen atoms, with the parameters vert max_sizevert and vert max_atomsvert set to 100.

### B. Chemical paths and interpolations

Suppose that exactly *t* characters differ in the corresponding indices of two SELFIES. Then there exist exactly *t*! paths between the two SELFIES. The length of all such paths is *t* as successive improvements are performed to the previous SELFIE string encountered in the path. Furthermore, similar to SMILES representations, a molecule can have multiple SELFIES representations allowing for multiple paths between any two given molecules. Considering *n* representations of the target structure, each of which has *e*_1_, *e*_2_, …, *e*_*n*_ corresponding starting SELFIE characters different, the total number of paths becomes 
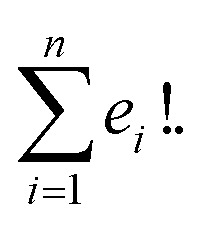


In Section II C 2, within a path, we randomly sampled molecules that necessarily increase fingerprint similarity allowing for the formation of a chemical path. log *P* and QED values of molecules in a path were estimated using RDKit.^48^ The docking scores were estimated with the SMINA open-source software^62^ using the setup proposed previously in the literature.^53^ Namely, the crystal structures for docking to 5-HT1B and CYP3D6 were obtained from the Protein Data Bank (PDB) (entry codes 4IAQ and 3QM4), the binding sites were selected manually, and the scores of the top 5 best-scoring binding poses were averaged to maximize consistency of the results. In both experiments, we considered different SMILES orderings of the starting and target molecules, respectively, and, between each pair, repeated the experiment several times, leading to different results, such that approximately 800 unique molecules from the paths were obtained. For path and chemical path formation between two SELFIES, we padded the string to the same length with a dummy character. The dummy character was removed from the SELFIES before converting to SMILES.

### C. Median molecules for photovoltaics

The molecules of the HCE database were ordered based on their ability to maximize one property, while minimizing the other simultaneously. The top 100 structures from this ordered list were selected for our experiment in Section II D. In the formation of generalized paths, the starting molecule is selected randomly and 10 000 paths were obtained between randomized orderings of the respective SMILES string. We ran semiempirical calculations to obtain the dipole moments, LUMO energies and HOMO–LUMO gaps for the HCE database and the top-100 unique median structures using GFN2-*x*TB.^55^ Random SELFIES for the experiment were generated *via* random combinations of the 34 SELFIEcharacters part of the semantically robust alphabet. The length of the generated random SELFIES was restricted to the largest number of characters within the SELFIES representations of the three reference molecules.

## Data availability statement

Full code is available at: https://github.com/aspuru-guzik-group/stoned-selfies.

## Author contributions

A. N. and M. K. conceived the initial idea of the project and performed first tests. The corresponding results were analyzed and discussed by all the authors. Based on these results, and with further ideas from A. N., R. P. and G. P. G., the project scope was expanded significantly. A. N. wrote the program code and performed all the computational experiments. All authors analyzed the corresponding results. The manuscript was mainly written by A. N. and R. P. with input from all other authors.

## Conflicts of interest

There are no conflicts to declare.

## Supplementary Material

SC-012-D1SC00231G-s001

## References

[cit1] Sanchez-Lengeling B., Aspuru-Guzik A. (2018). Inverse molecular design using machine learning: Generative models for matter engineering. Science.

[cit2] Kingma D. P. and Welling M., Auto-encoding variational bayes, 2013, arXiv preprint arXiv:1312.6114

[cit3] Gómez-Bombarelli R., Wei J. N., Duvenaud D., Hernández-Lobato J. M., Sánchez-Lengeling B., Sheberla D., Aguilera-Iparraguirre J., Hirzel T. D., Adams R. P., Aspuru-Guzik A. (2018). Automatic chemical design using a data-driven continuous representation of molecules. ACS Cent. Sci..

[cit4] Goodfellow I., Pouget-Abadie J., Mirza M., Xu B., Warde-Farley D., Ozair S., Courville A., and Bengio Y., Generative adversarial nets, in Advances in neural information processing systems, 2014, pp. 2672–2680

[cit5] De Cao N. and Kipf T., Molgan: An implicit generative model for small molecular graphs, 2018, arXiv preprint arXiv:1805.11973

[cit6] Sutskever I., Vinyals O., and Quoc V. L., Sequence to sequence learning with neural networks, in Advances in neural information processing systems, 2014, pp. 3104–3112

[cit7] Marwin H. S. S., Kogej T., Tyrchan C., Waller M. P. (2018). Generating focused molecule libraries for drug discovery with recurrent neural networks. ACS Cent. Sci..

[cit8] Li Y., Deep reinforcement learning: An overview, 2017, arXiv preprint arXiv:1701.07274

[cit9] Neil D., Segler M. H. S., Guasch L., Ahmed M., Dean P., Sellwood M., and Brown N., Exploring deep recurrent models with reinforcement learning for molecule design. in ICLR, 2018, https://openreview.net/forum?id=Bk0xiI1Dz

[cit10] Westhead D. R., Clark D. E., Frenkel D., Jin Li, Murray C. W., Robson B., Pro_ligand B. W. (1995). An approach to de novo molecular design. 3. a genetic algorithm for structure refinement. J. Comput.-Aided Mol. Des..

[cit11] Glen R. C., Payne A. W. R. (1995). A genetic algorithm for the automated generation of molecules within constraints. J. Comput.-Aided Mol. Des..

[cit12] Dominique D., Thoreau E., Grassy G. (2000). A genetic algorithm for the automated generation of small organic molecules: Drug design using an evolutionary algorithm. J. Comput.-Aided Mol. Des..

[cit13] Vasundhara Devi R., Siva Sathya S., Selvaraj Coumar M. (2015). Evolutionary algorithms for de novo drug design–a survey. Appl. Soft Comput..

[cit14] Jensen J. H. (2019). A graph-based genetic algorithm and generative model/monte carlo tree search for the exploration of chemical space. Chem. Sci..

[cit15] Yoshikawa N., Terayama K., Sumita M., Homma T., Oono K., Tsuda K. (2018). Population-based de novo molecule generation, using grammatical evolution. Chem. Lett..

[cit16] Hoksza D., Škoda P., Voršilák M., Svozil D. (2014). Molpher: a software framework for systematic chemical space exploration. J. Cheminf..

[cit17] Reeves S., Benjamin DiF., Shahani V., MacKinnon S., Windemuth A., Brereton A. E. (2020). Assessing methods and obstacles in chemical space exploration. Applied AI Letters.

[cit18] Weininger D. (1988). Smiles, a chemical language and information system. 1. introduction to methodology and encoding rules. J. Chem. Inf. Comput. Sci..

[cit19] Kwon Y. and Lee J.. Molfinder: An efficient global molecular property optimization and search algorithm using smiles, ChemRxiv, 2020, https://chemrxiv.org/articles/preprint/MolFinder_An_Efficient_Global_Molecular_Property_Optimization_and_Search_Algorithm_Using_SMILES/13106891/1

[cit20] Jin W., Barzilay R., and Jaakkola T., Junction tree variational autoencoder for molecular graph generation, 2018, arXiv preprint arXiv:1802.04364

[cit21] You J., Liu B., Ying Z., Pande V., and Leskovec J., Graph convolutional policy network for goal-directed molecular graph generation, in Advances in neural information processing systems, 2018, pp. 6410–6421

[cit22] Krenn M., Häse F., Nigam A. K., Friederich P., and Aspuru-Guzik A., Self-referencing embedded strings (selfies): A 100% robust molecular string representation, 2019, arXiv preprint arXiv:1905.13741

[cit23] Nigam A. K., Friederich P., Krenn M., and Aspuru-Guzik A., Augmenting genetic algorithms with deep neural networks for exploring the chemical space, 2019, arXiv preprint arXiv:1909.11655

[cit24] van Deursen R., Reymond J.-L. (2007). Chemical space travel. ChemMedChem.

[cit25] Potyrailo R., Rajan K., Klaus S., Takeuchi I., Chisholm B., Lam H. (2011). Combinatorial and high-throughput screening of materials libraries: review of state of the art. ACS Comb. Sci..

[cit26] dos Passos Gomes G., Pollice R., Aspuru-Guzik A. (2021). Navigating through the maze of homogeneous catalyst design with machine learning. Trends Chem..

[cit27] Zoete V., Grosdidier A., Michielin O. (2009). Docking, virtual high throughput screening and in silico fragment-based drug design. J. Cell. Mol. Med..

[cit28] Bender A., Glen R. C. (2004). Molecular similarity: a key technique in molecular informatics. Org. Biomol. Chem..

[cit29] Eckert H., Bajorath J. (2007). Molecular similarity analysis in virtual screening: foundations, limitations and novel approaches. Drug discovery today.

[cit30] Gordon E. M., W Barrett R., Dower W. J., Fodor S. P. A., Gallop M. A. (1994). Applications of combinatorial technologies to drug discovery. 2. combinatorial organic synthesis, library screening strategies, and future directions. J. Med. Chem..

[cit31] Hachmann J., Olivares-Amaya R., Atahan-Evrenk S., Amador-Bedolla C., Sánchez-Carrera R. S., Gold-Parker A., Vogt L., Anna M. B., Aspuru-Guzik A. (2011). The harvard clean energy project: large-scale computational screening and design of organic photovoltaics on the world community grid. J. Phys. Chem. Lett..

[cit32] Brown N., Fiscato M., Marwin H. S. S., Vaucher A. C. (2019). Guacamol: benchmarking models for de novo molecular design. J. Chem. Inf. Model..

[cit33] Polykovskiy D., Alexander Z., Sanchez-Lengeling B., Golovanov S., Tatanov O., Belyaev S., Kurbanov R., Artamonov A., Aladinskiy V. and Veselov M., *et al.*, Molecular sets (moses): a benchmarking platform for molecular generation models, 2018, arXiv preprint arXiv:1811.12823 10.3389/fphar.2020.565644 PMC7775580 33390943

[cit34] Clemett D., Goa K. L. (2000). Celecoxib. Drugs.

[cit35] Polishchuk P. (2020). Crem: chemically reasonable mutations framework for structure generation. J. Cheminf..

[cit36] Davies M., Nowotka M., George P., Dedman N., Gaulton A., Atkinson F., Bellis L., Overington J. P. (2015). ChEMBL web services: streamlining access to drug discovery data and utilities. Nucleic Acids Res..

[cit37] Gaulton A., Hersey A., Michał Nowotka A. (2016). Patrícia Bento, Jon Chambers, David Mendez, Prudence Mutowo, Francis Atkinson, Louisa J. Bellis, Elena Cibrián-Uhalte, Mark Davies, Nathan Dedman, Anneli Karlsson, María Paula Magariños, John P. Overington, George Papadatos, Ines Smit, and Andrew R. Leach. The ChEMBL database in 2017. Nucleic Acids Res..

[cit38] Seth D. A., Huang Xi-P., Cáceres E. L., Gendelev L., Roth B. L., Keiser M. J. (2017). A simple representation of three-dimensional molecular structure. J. Med. Chem..

[cit39] Todeschini R. and Consonni V., Handbook of molecular descriptors, John Wiley & Sons, 2008, vol. 11

[cit40] Graziano G. (2020). Fingerprints of molecular reactivity. Nat. Rev. Chem..

[cit41] Cano G., Garcia-Rodriguez J., Garcia-Garcia A., Perez-Sanchez H., Benediktsson J. A., Thapa A., Barr A. (2017). Automatic selection of molecular descriptors using random forest: Application to drug discovery. Expert Syst. Appl..

[cit42] Brown N., McKay B., Gilardoni F., Gasteiger J. (2004). A graph-based genetic algorithm and its application to the multiobjective evolution of median molecules. J. Chem. Inf. Comput. Sci..

[cit43] Jiang X., Munger A., Bunke H. (2001). An median graphs: properties, algorithms, and applications. IEEE Trans. Pattern Anal. Mach. Intell..

[cit44] Henault E. S., Rasmussen M. H., Jensen J. H. (2020). Chemical space exploration: how genetic algorithms find the needle in the haystack. PeerJ Physical Chemistry. PeerJ Physical Chemistry.

[cit45] Noel O. 'B. and Dalke A., Deepsmiles: An adaptation of smiles for use in machine-learing chemical structures, ChemRxiv, 2018, https://chemrxiv.org/articles/preprint/DeepSMILES_An_Adaptation_of_SMILES_for_Use_in_Machine-Learning_of_Chemical_Structures/7097960/1

[cit46] Scott A. W., Gordon M. C. (1999). Prediction of physicochemical parameters by atomic contributions. J. Chem. Inf. Comput. Sci..

[cit47] Richard Bickerton G., Gaia V. P., Besnard J., Muresan S., Hopkins A. L. (2012). Quantifying the chemical beauty of drugs. Nat. Chem..

[cit48] Landrum G., *et al.*, Rdkit: Open-source cheminformatics, 2006

[cit49] Pantsar T., Poso A. (2018). Binding affinity via docking: fact and fiction. Molecules.

[cit50] Wang C., Jiang Y., Ma J., Wu H., Wacker D., Katritch V., Han G. W., Liu W., Huang Xi-P., Vardy E. (2013). *et al.*, Structural basis for molecular recognition at serotonin receptors. Science.

[cit51] Wang A., Savas U., Hsu M.-H., Stout C. D., Johnson E. F. (2012). Crystal structure of human cytochrome p450 2d6 with prinomastat bound. J. Biol. Chem..

[cit52] Teh L. K. and Bertilsson L., Pharmacogenomics of cyp2d6: molecular genetics, interethnic differences and clinical importance, Drug metabolism and pharmacokinetics, 2011, pp. 1112190300–1112190300 10.2133/dmpk.dmpk-11-rv-121 22185816

[cit53] Cieplinski T., Danel T., Podlewska S., and Jastrzebski S., We should at least be able to design molecules that dock well, 2020, arXiv preprint arXiv:2006.16955 10.1021/acs.jcim.2c01355 PMC10268949 37224003

[cit54] Häse F., Loïc M. R., Friederich P., Aspuru-Guzik A. (2020). Designing and understanding light-harvesting devices with machine learning. Nat. Commun..

[cit55] Bannwarth C., Ehlert S., Grimme S. (2019). Gfn2-xtb—an accurate and broadly parametrized self-consistent tight-binding quantum chemical method with multipole electrostatics and density-dependent dispersion contributions. J. Chem. Theory Comput..

[cit56] Bredt J. (1924). Über sterische hinderung in brückenringen (bredtsche regel) und über die meso-trans-stellung in kondensierten ringsystemen des hexamethylens. Justus Liebigs Ann. Chem..

[cit57] Brown N., McKay B., Gasteiger J. (2004). The de novo design of median molecules within a property range of interest. J. Comput.-Aided Mol. Des..

[cit58] Jonas V., Van den Abeele J. (2020). Illuminating elite patches of chemical space. Chem. Sci..

[cit59] Pyzer-Knapp E. O., Suh C., Gómez-Bombarelli R., Aguilera-Iparraguirre J., Aspuru-Guzik A. (2015). What is high-throughput virtual screening? a perspective from organic materials discovery. Annu. Rev. Mater. Res..

[cit60] Renz P., Van Rompaey D., Wegner J. K., Hochreiter S., Klambauer G. (2019). On failure modes in molecule generation and optimization. Drug Discovery Today: Technol..

[cit61] https://github.com/DrrDom/crem, March 2021

[cit62] Koes D. R., Baumgartner M. P., J Camacho C. (2013). Lessons learned in empirical scoring with smina from the csar 2011 benchmarking exercise. J. Chem. Inf. Model..

