# Supplementary material for: Beyond generative models: superfast traversal, optimization, novelty, exploration and discovery (STONED) algorithm for molecules using SELFIES
Source: Chem Sci. 2021 Apr 20;12(20):7079–90. doi: 10.1039/d1sc00231g (PMC8153210; doi:10.1039/d1sc00231g)
Supplement: SC-012-D1SC00231G-s001 [file SC-012-D1SC00231G-s001.pdf]

Electronic Supplementary Material (ESI) for Chemical Science.
This journal is © The Royal Society of Chemistry 2021

Supplementary Information:
Efficient Interpolation and Exploration in the Chemical Space Using String Representations

S1. FORMATION OF LOCAL CHEMICAL SPACES

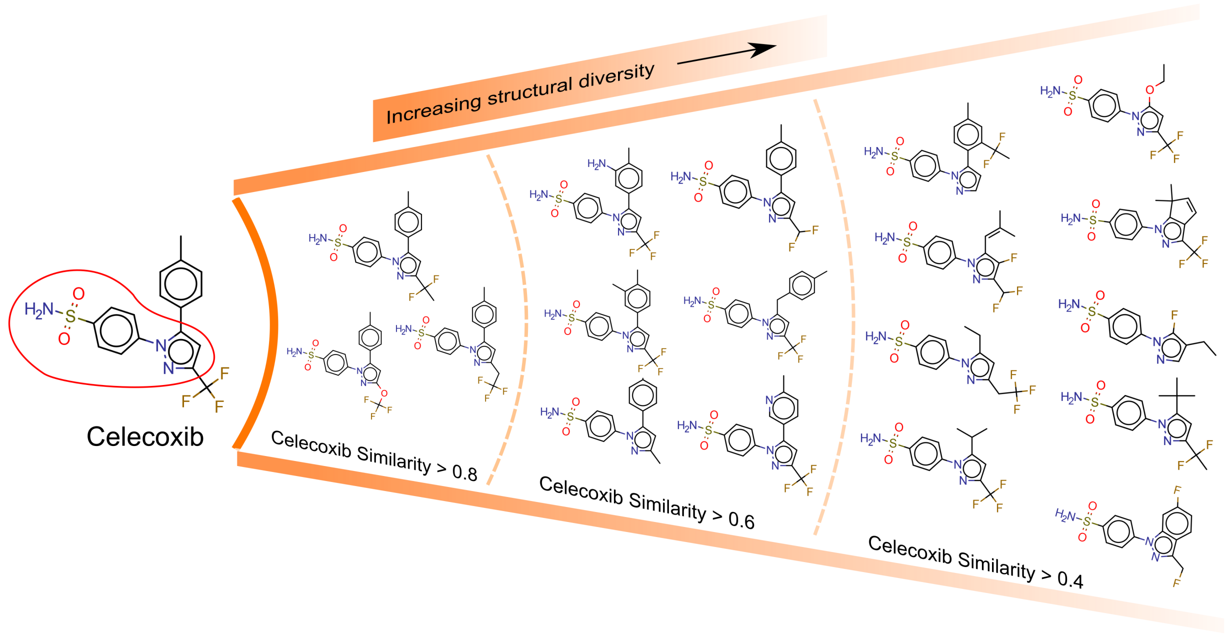

FIG. S1. Illustration of string manipulations within STONED to form local chemical subspaces (Section II B), while preserving a scaffold of Celecoxib. Shown molecules correspond to the scaffold constraint experiment of Table I. The selected preserved scaffold is circled in red.

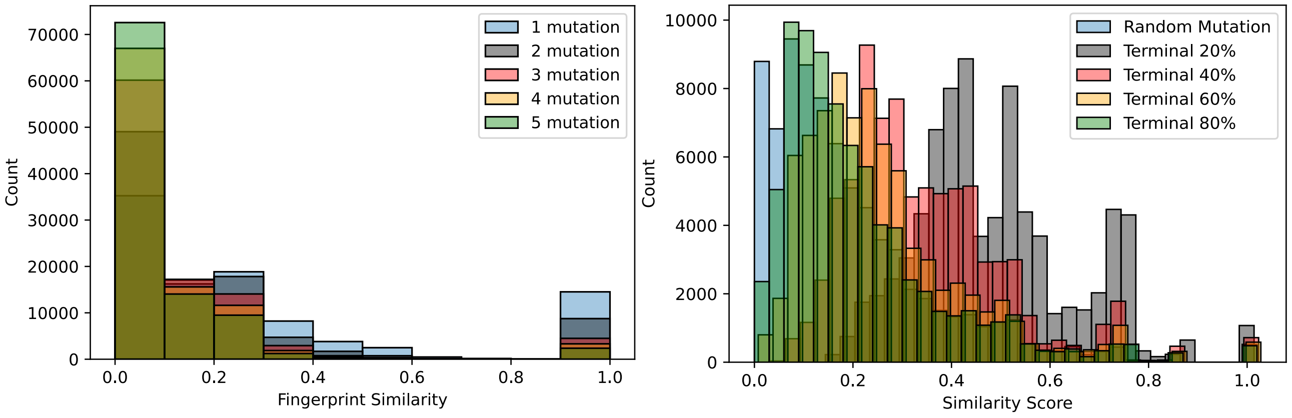

FIG. S2. Analysis of the effect of random SELFIE string mutations on molecular similarity. (Left): Up to 5 random mutations are performed on random molecules (i.e., randomly generated SELFIES), and the Tanimoto similarity between the ECFP4 fingerprints of the initial and mutated molecules is calculated. (Right) Random SELFIE string mutations of Celecoxib are restricted to the terminal 20-80 % of the SELFIE characters.

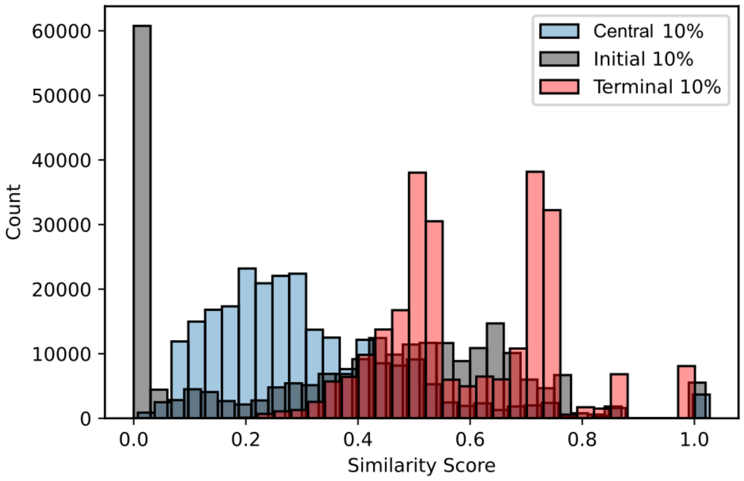

FIG. S3. Random SELFIE string mutations of Celecoxib are restricted to the central, initial or terminal 10% of the SELFIE characters.

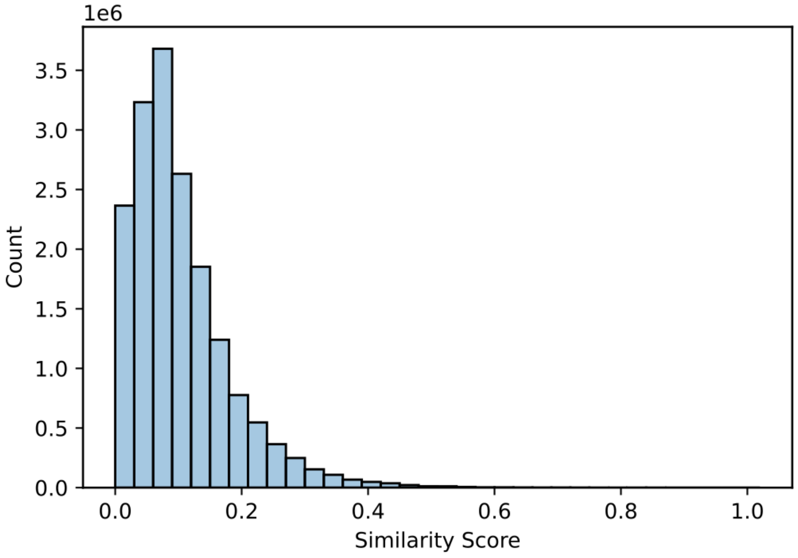

FIG. S4. Distribution of the fingerprint similarity with respect Celecoxib of the structures generated in the local chemical subspace by performing mutations on the next nearest neighbors.

S2. ANALYSIS OF JOINT SIMILARITY FUNCTIONS

We begin by analyzing the use of the geometric mean for measuring join similarity, as suggested in the GuacaMol benchmark tasks, for molecule m, with respect to molecules m' and m'. Say sim$(m,\, m') = 0.1$, and sim$(m,\, m'') = 0.9$, then the geometric mean is 0.3. Alternatively, if sim$(m,\, m') = 0.3$, and sim$(m,\, m'') = 0.3$, the geometric mean is 0.3 as well. Naturally, the molecule in the first example is more biased to just one of the structures, while in the second example the structure is more representative of both. We illustrate the values of the geometric mean of the molecular similarities for the cases of two and three reference molecules in Figure S5(b). This problem becomes more prominent when the arithmetic mean (see Figure S5(a)) is used instead of the geometric mean – in cases where m is the same as m' or m'', and there is no similarity between m' and m'', the score trivially reaches 0.5. These observations motivated our development of Equation 1 (Figure S5(c)).

Equation 1 shows the following boundary conditions:

1. When molecule m is perfectly similar to all the molecules of the set $M = m', m'', ...$, F(m) computes to 1.
2. When the molecule m is similar to none of the structures of M, F(m) computes to 0.
3. When the molecule is similar to only one structure in M, the minimum of the function is achieved, because all

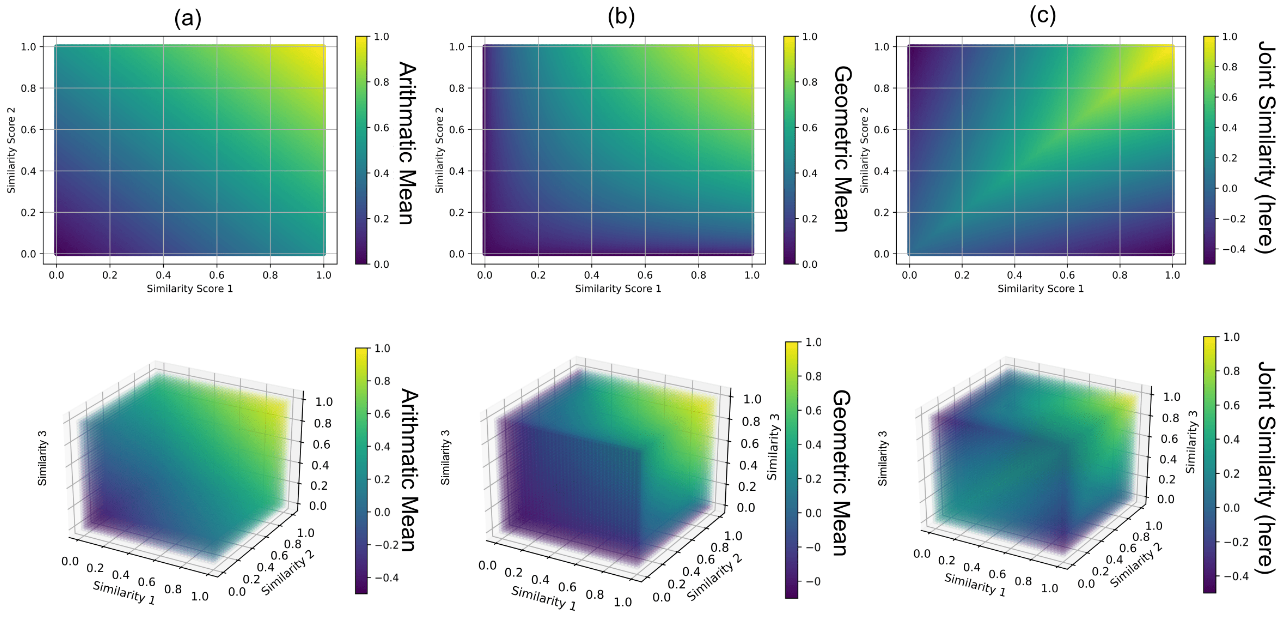

FIG. S5. Joint molecular similarity, calculated with (a) arithmetic mean, (b) geometric mean, and (c) Equation 1 for a set of two (top row) and three structures (bottom row). The axes indicate the Tanimoto similarity of the extended connectivity fingerprints, between a molecule and a reference structure, within the set.

similarity scores range from 0 to 1. The value is obtained as:

$$F(m) = \frac{1}{n} - 1 = \frac{1}{n} - \frac{n}{n} = \frac{1-n}{n}, \text{ where } n = |m| \tag{2}$$

For intuitiveness, we fit a third order polynomial through the newly defined joint similarity F(m) with the above three values (i.e., 0, 1, and $\frac{1-n}{n}$) as input values and assign the target values 0, 1, and -1 to them, respectively. Consequently, we observe an increasing gradual movement from:

1. Similar to only one structure in M: Joint similarity of -1.
2. Similar to none or strong resemblance to only a few structures: Joint similarity close to 0.
3. Similar to all molecules: Joint similarity of 1.

Consequently, values larger than 0 indicate molecules that are truly similar to all reference molecules. The polynomial can be computed on the fly depending on the number of reference molecules n and is uniquely defined by the three boundary conditions explained above and by imposing a local maximum at the point (1,1).

S3. CHEMICAL PATH FORMATION AND GENERALIZED CHEMICAL PATHS

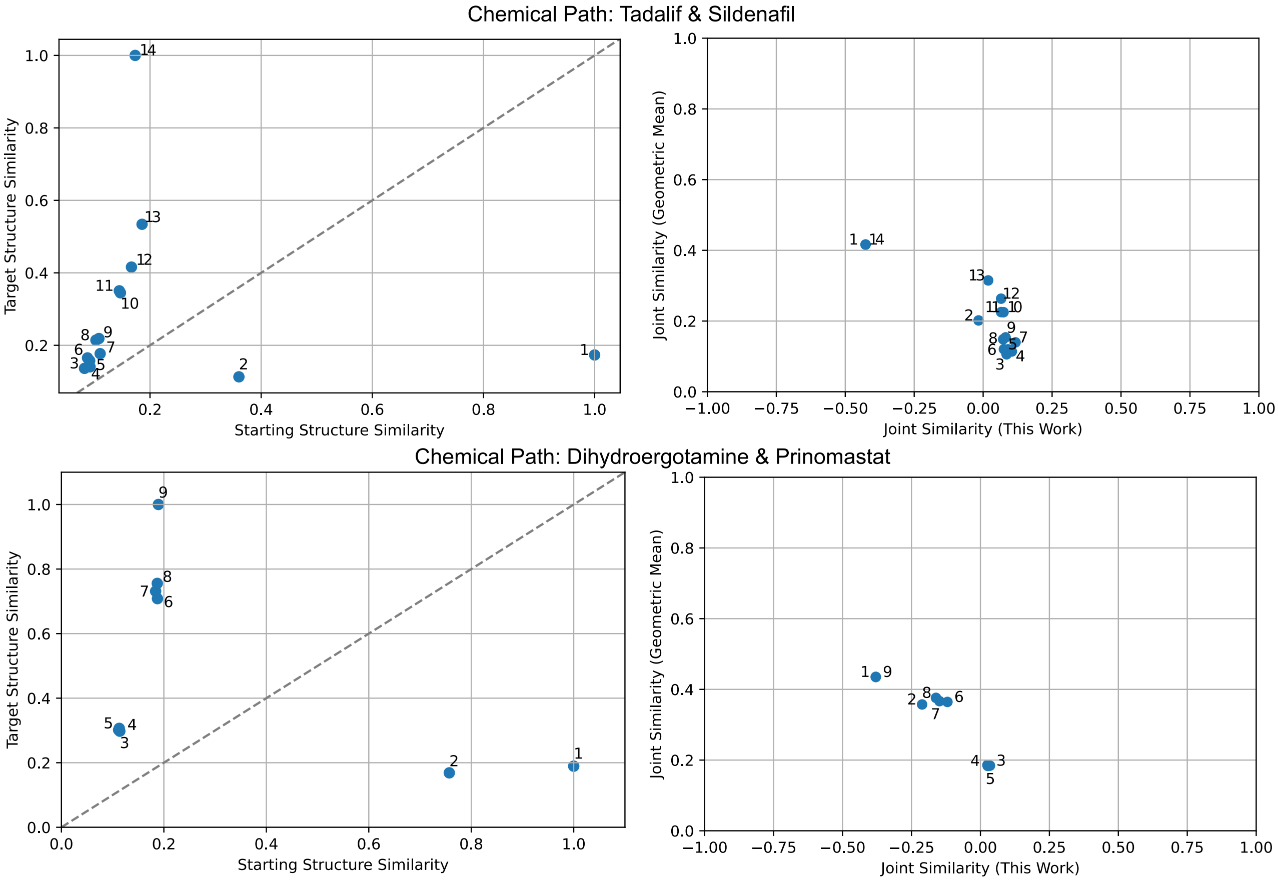

FIG. S6. Similarities of molecules along chemical paths to both the starting and the target structures (left) and comparison of the geometric mean joint similarity to our newly defined joint similarity along chemical paths (right). (Top) Selected chemical path between Tadalif and Sildenafil. (Bottom) Selected chemical path between Dihydroergotamine and Prinomastat.

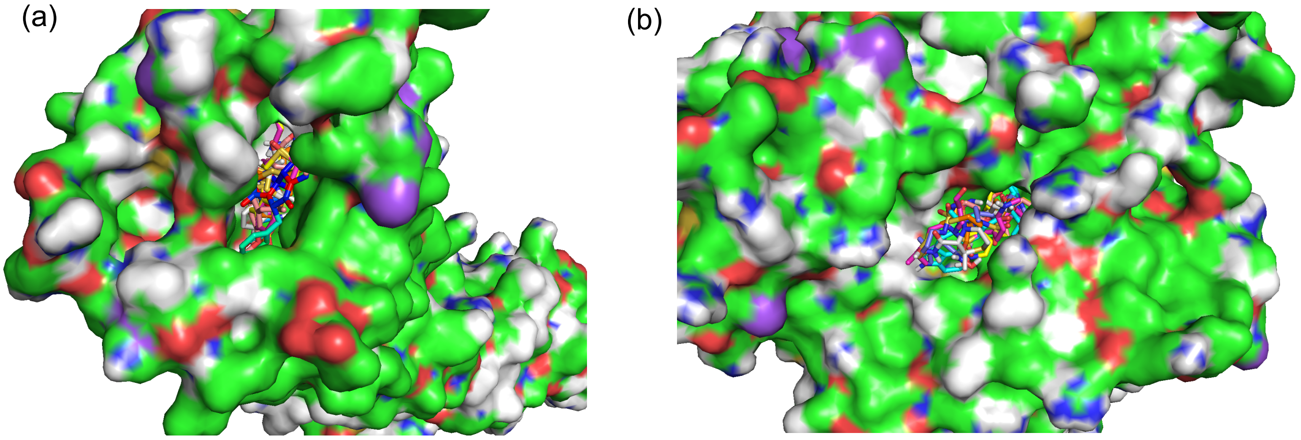

FIG. S7. Docked molecules along the chemical path between Dihydroergotamine and Prinomastat shown Figure 5. (a) Generated molecules are docked onto 5-HT1B, and (b) onto CYP2D6.

To form a generalized path between a molecule m, and a set of molecules M, we randomly select an index i in the SELFIES representation of m and perform distinct mutations, yielding $|M|$ different SELFIES. The distinct strings are obtained by selecting the i-th character within the SELFIES representing the molecules of M, and mutating the SELFIES character at index i in m to the one of the corresponding target characters. Among these $|M|$ distinct

SELFIES, the joint similarity is calculated after conversion to SMILES, and the molecule with the largest joint similarity is identified. The process is repeated with this new SELFIES until all distinct indices are covered.

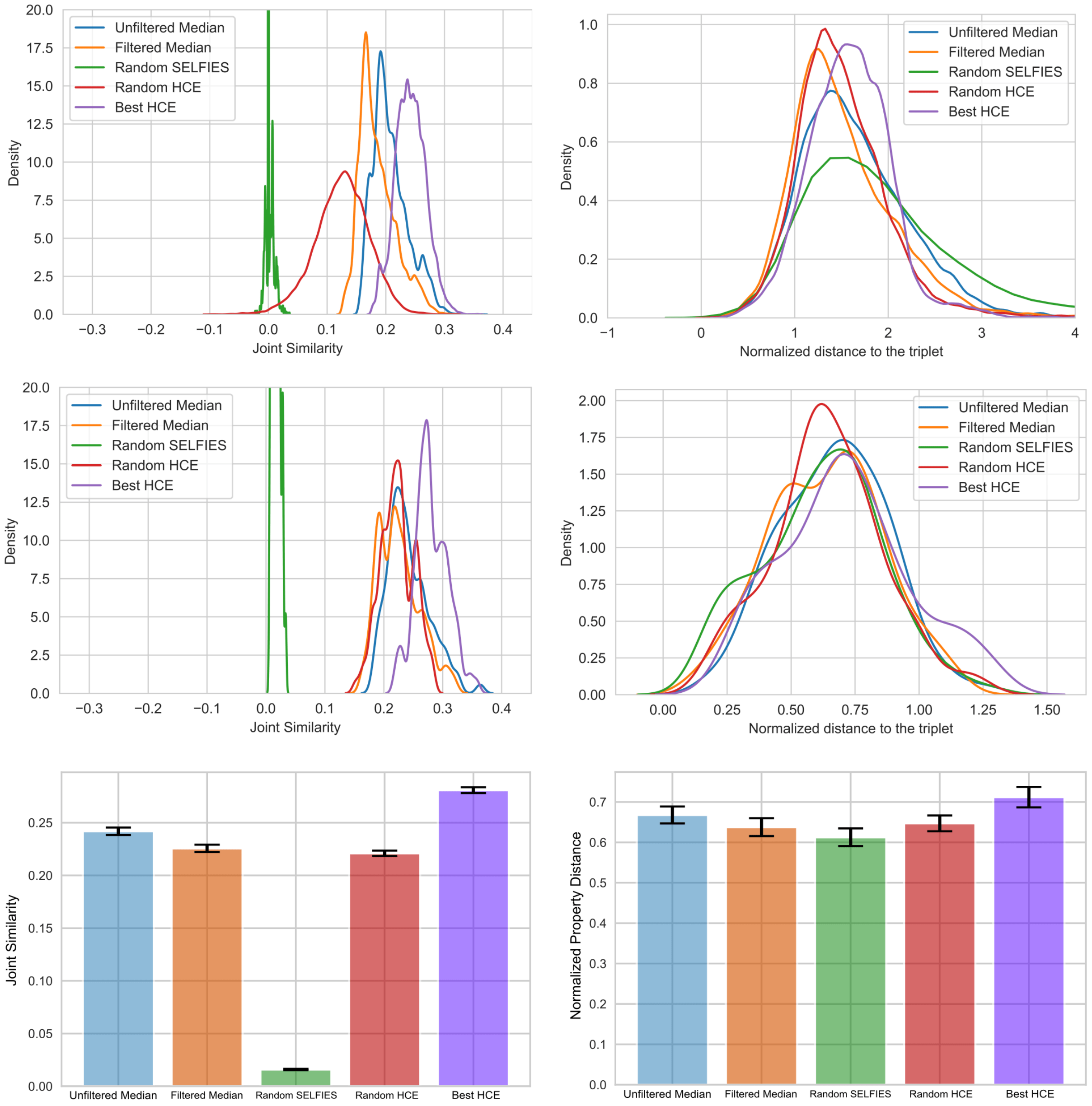

FIG. S8. Structural (left) and property similarity (right) of generated median molecules compared to specific triplets of molecules collected from the Harvard Clean Energy database. Density plots are shown for the joint similarity and the normalized property distance of the best median structures, for the best 100 medians (top row) and the top median (middle row). Bar plots for the mean, and error bars for the standard deviation of the mean (two standard deviations are depicted) are shown for the joint similarity and the normalized property distance of the best median structure (bottom row).

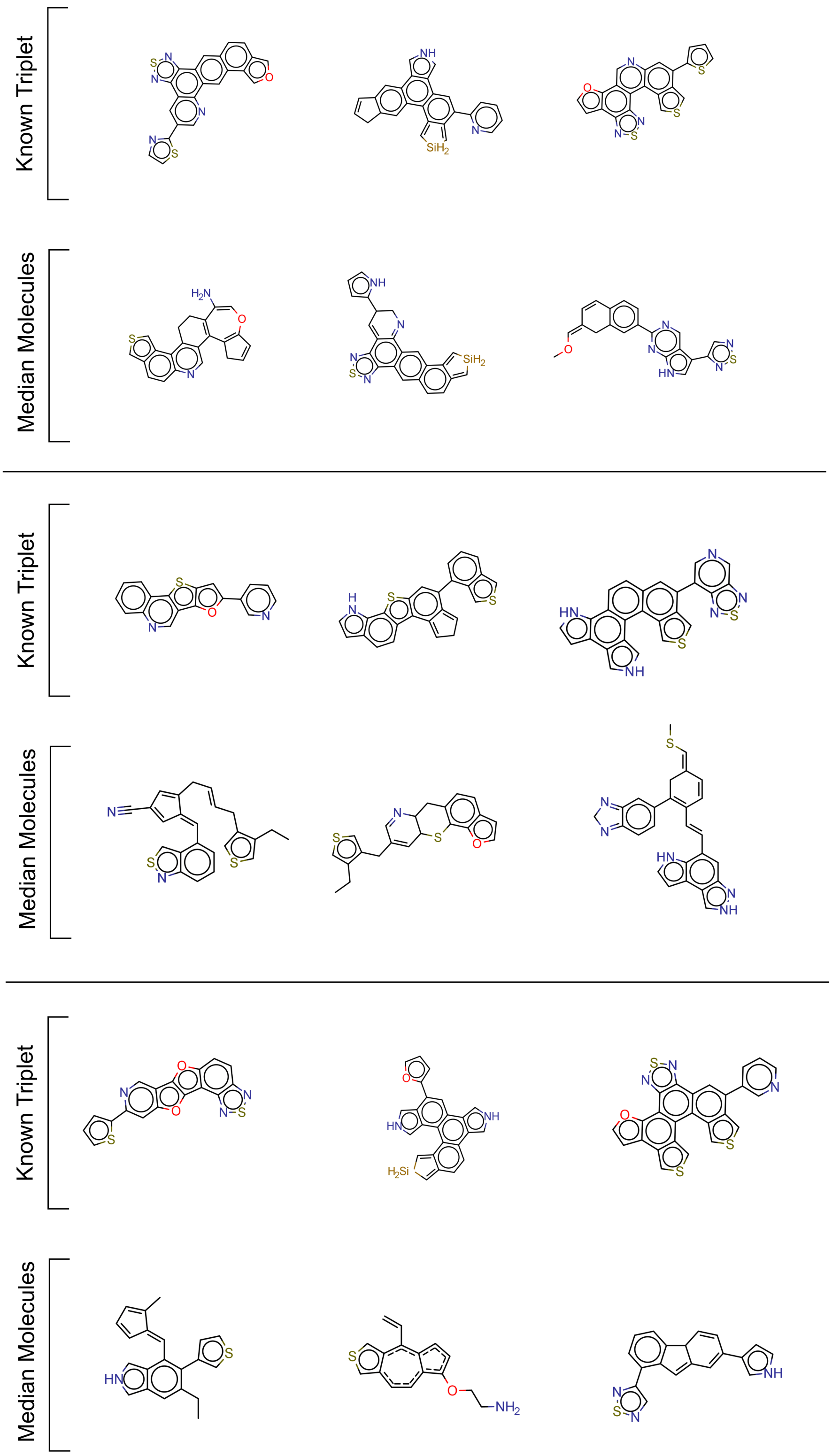

FIG. S9. Filtered median molecules and their starting triplets for Section II D.

TABLE S3. Joint similarity and normalized property distance of the 100 most similar medians produced on 100 triplets of the Harvard Clean Energy benchmark introduced in Section 2.4. Mean and standard deviation of the mean are provided.

	**Top Median**		**Top 100 Medians**	
	Joint Similarity	Normalized distance	Joint Similarity	Normalized distance
Unfiltered Median	0.242 ± 0.0035	0.668 ± 0.0210	0.242 ± 0.0035	1.648 ± 0.0061
Filtered Median	0.226 ± 0.0035	0.638 ± 0.0221	0.186 ± 0.0003	1.532 ± 0.0061
Random SELFIES	0.017 ± 0.0006	0.633 ± 0.0236	0.000 ± 0.0000	2.174 ± 0.0198
Random HCE	0.222 ± 0.0026	0.646 ± 0.0221	0.126 ± 0.0005	1.516 ± 0.0056
Best HCE	0.281 ± 0.0028	0.712 ± 0.0253	0.242 ± 0.0003	1.587 ± 0.0045

S4. COMPARISON OF MOLECULE GENERATION ALGORITHMS

TABLE S4. Performance of STONED on the GuacaMol benchmark tasks together with the performance of baseline models from the literature [32, 35].

**Benchmark**	**STONED**	**SMILES GA**	**SMILES LSTM**	**CReM**	**Graph GA**
Celecoxib rediscovery	0.556	0.732	1.000	1.000	1.000
Troglitazone rediscovery	0.543	0.515	1.000	1.000	1.000
Thiothixene rediscovery	0.677	0.598	1.000	1.000	1.000
Aripiprazole similarity	0.716	0.834	1.000	1.000	1.000
Albuterol similarity	0.939	0.907	1.000	1.000	1.000
Mestranol similarity	0.769	0.790	1.000	1.000	1.000
C11H24	1.000	0.829	0.993	0.966	0.971
C9H10N2O2PF2Cl	0.886	0.889	0.879	0.940	0.982
Median molecules 1	0.351	0.334	0.438	0.371	0.406
Median molecules 2	0.395	0.380	0.422	0.434	0.432
Osimertinib MPO	0.863	0.886	0.907	0.995	0.953
Fexofenadine MPO	0.878	0.931	0.959	1.000	0.998
Ranolazine MPO	0.812	0.881	0.855	0.969	0.920
Perindopril MPO	0.629	0.661	0.808	0.815	0.792
Amlodipine MPO	0.738	0.722	0.894	0.902	0.894
Sitagliptin MPO	0.592	0.689	0.545	0.763	0.891
Zaleplon MPO	0.674	0.413	0.669	0.770	0.754
Valsartan SMARTS	0.864	0.552	0.978	0.994	0.990
Deco Hop	0.968	0.970	0.996	1.000	1.000
Scafold Hop	0.854	0.885	0.998	1.000	1.000
Total Score	14.704	14.396	17.340	17.919	17.983
